# A semi-automated protocol for Archaea DNA extraction from stools

**DOI:** 10.1186/1756-0500-6-186

**Published:** 2013-05-07

**Authors:** Saber Khelaifia, Pierre-Yves Ramonet, Marielle Bedotto Buffet, Michel Drancourt

**Affiliations:** 1 Aix Marseille Université, URMITE, UMR63 CNRS 7278, IRD 198, Inserm 1095, 13005, Marseille, France; 2 Unité des Rickettsies, Faculté de Médecine, 27, Boulevard Jean Moulin, Cedex 5, Marseille, France

**Keywords:** Human-associated archaea, Methanogenic archaea, Microbiota, DNA extraction, Archaeal DNA

## Abstract

**Background:**

The PCR-based detection of archaea DNA in human specimens relies on efficient DNA extraction. We previously designed one such protocol involving only manual steps. In an effort to reduce the workload involved, we compared this manual protocol to semi-automated and automated protocols for archaea DNA extraction from human specimens.

**Findings:**

We tested 110 human stool specimens using each protocol. An automated protocol using the EZ1 Advanced XL extractor with the V 1.066069118 Qiagen DNA bacteria card and the EZ1® DNA Tissue Kit (Qiagen, Courtaboeuf, France) yielded 35/110 (32%) positives for the real-time PCR detection of the *Methanobrevibacter smithii* 16S rRNA gene, with average Ct values of 36.1. A semi-automated protocol combining glass-powder crushing, overnight proteinase K digestion and lysis in the buffer from the EZ1 kit yielded 90/110 (82%) positive specimens (*P* = 0.001) with an average Ct value of 27.4 (*P* = 0.001). The manual protocol yielded 100/110 (91%) positive specimens (*P* = 0.001) with an average Ct value of 30.33 (*P* = 0.001). However, neither the number of positive specimens nor the Ct values were significantly different between the manual protocol and the semi-automated protocol (*P* > 0.1 and *P* > 0.1).

**Conclusion:**

Proteinase K digestion and glass powder crushing dramatically increase the extraction yield of archaea DNA from human stools. The semi-automated protocol described here was more rapid than the manual protocol and yielded significantly more archaeal DNA. It could be applied for extracting total stool DNA for further PCR amplification.

## Findings

Archaea are permanent inhabitants of the human gut. Three methanogenic species, *Methanobrevibacter smithii *[1], *Methanosphaera stadtmanae *[2] and *Methanomassiliicoccus luminyensis *[3,4], have been isolated from human stools, and *Methanobrevibacter oralis* has been isolated from the subgingival plaque [4,5]. In addition to their role in the local homeostasis of anaerobic communities [6], methanogenic archaea are suspected to be involved in digestive tract diseases and obesity [7-9] and have been implicated in periodontitis [10-12]. In addition to fastidious isolation and culture, PCR-based techniques have provided additional information about cultured archaea [6,13] and further revealed the presence of as-yet uncultured archaea [3,13,14]. Several different approaches have been used to extract DNA from human feces [13-15], and various methods have been described [16-18]. We previously showed that an appropriate extraction protocol increased the archaeal DNA yield from human stools [14]. However, this protocol involved only manual steps, making it too labor intensive for routine diagnostic use. We therefore aimed to reduce the number of manual steps and compared automated, semi-automated and reference manual DNA extraction protocols for the real-time PCR detection of *M. smithii* in human feces sample.

This study included 110 stool specimens prospectively collected in 110 individuals from Marseille, France, between July and August 2011 as a part of routine diagnostic activity in the Microbiology laboratory, Timone Hospital, Méditerranée Infection, Marseille, France. No written consent was needed for this work in accordance with the “LOI n° 2004-800 relative à la bioéthique” published in the “Journal Officiel de la République Française” the 6 August 2004 since no additional sample was taken for the study. According to this law, patients were informed that stool specimens could be used for anonymised study. This study was approved by the local Ethics Committee IFR48. Three different DNA extraction protocols were performed in parallel. The reference manual protocol using the NucleoSpin® Tissue Mini Kit (Macherey Nagel, Hoerdt, France) was performed as previously described [15]. The automated protocol involved DNA extraction using the EZ1 Advanced XL extractor with the V 1.066069118 Qiagen DNA bacteria card and the EZ1® DNA Tissue Kit (Qiagen, Courtaboeuf, France) as described by the manufacturer. A semi-automated protocol was performed as follows: approximately 1 gram of stool specimen was suspended in 5 mL Tris-HCl 0.05 M, pH 7.5. A 250 μL aliquot of the suspension was transferred to a sterile screw-cap Eppendorf tube containing 0.3 g of acid-washed beads (≤106 mm; Sigma, Saint-Quentin-Fallavier, France) and shaken in a FastPrep BIO 101 apparatus (Qbiogene, Strasbourg, France) at level 6.5 (full speed) for 90 s to achieve mechanical lysis. The supernatant was collected and incubated overnight at 56°C with 180 μL of lysis buffer and 25 μL proteinase K (20 mg/mL) from the Qiagen EZ1® DNA Tissue Kit. After a second cycle of mechanical lysis, the supernatant was incubated for 10 min at 100°C, and total DNA was then extracted using the Qiagen EZ1® DNA Tissue Kit in the EZ1 Advanced XL extractor with the V 1.066069118 Qiagen DNA bacteria card. Negative controls consisting of sterile DNA-free water were introduced at all steps and underwent the same extraction process that was used for the stool specimens. The working time required for each protocol was measured on three separate occasions.

Extracted total DNA was used as a template for the real-time PCR detection of the *M. smithii* 16S rRNA gene using PCR primers Smit.16S-740F: 5′-CCGGGTATCTAATCCGGTTC-3′ and Smit.16S-862R: 5′-CTCCCAGGGTAGAGGTGAAA-3′ and the probe Smit.16S FAM: 5′-CCGTCAGAATCGTTCCAGTCAG-3′, adapted from a previously described protocol [15]. A quantification synthetic plasmid was used as an internal control to monitor PCR inhibition; total bacterial load was measured a previously described [15]. Real time-PCR products were sequenced using the primers Smit.16S-740F, Smit.16S-862R, the BigDye Terminator 1.1 Cycle Sequencing kit and the 3130 Genetic Analyzer (Applied Biosystems, Villebon sur Yvette, France). Negative controls were incorporated into each assay. Sequences were analyzed using the Seqscape program (Applied Biosystems), and sequence similarity values were determined using the online BLAST program at NCBI (http://www.ncbi.nlm.nih.gov/BLAST/).

The negative controls remained negative in all experiments, and no archaeal DNA was extracted from the water used as negative control. The internal plasmid control was detected in all specimens, with median Ct value of 24.6 for automated protocol; of 23.9 for semi-automated protocol and of 23.6 for manual protocol. Likewise, all bacteria detection was positive in all specimens with respective Ct value of 32.1, 25.9 and 26.4. These data indicated the absence of PCR inhibition in any of the tested protocols. All real time-PCR positive product sequences had 99% sequence similarity to the *M. smithii* reference sequence (GenBank accession number CP000678). This test was used as a positive control for real-time PCR detection. We compared three microbial DNA extraction protocols to identify an optimized protocol to obtain archaea DNA from human fecal specimens (Table 1). The automated protocol based on the EZ1 Advanced XL extractor and EZ1® DNA Tissue Kit yielded 35/110 (32%) positive specimens with an average Ct value of 36.1 ± 6.23, while the semi-automated protocol combining the EZ1® DNA Tissue Kit with mechanical disruption and enzymatic and chemical digestion yielded 90/110 (87%) positive specimens with an average cycle threshold (Ct) Ct value of 27.4 ± 7.1. Compared with the automated protocol, the semi-automated protocol yielded significantly more positive specimens (*P* < 0.001; Student’s t test) and significantly lower Ct values (*P* < 0.001). The previously described manual protocol [15] yielded 100/110 (91%) positive specimens with an average Ct value of 30.33 ± 6.36. The manual protocol yielded significantly more positive specimens and significantly lower Ct values than the automated protocol (*P* < 0.001 and *P* < 0.001, respectively); however, neither the number of positive specimens nor the Ct values were significantly different between the manual protocol and the semi-automated protocol (*P* > 0.1 and *P* > 0.1).

**Table 1:** Statistical analysis applied to 110 human specimens extracted using three DNA extraction methods

	**Manual protocol**	**Semi-automated**	**Automated protocol**
Positive specimens (%)	100 (91%)	90 (87%)	35 (32%)
Negative specimens	10	20	75
Average, Ct	30.33	27.4	36.1
Standard deviation, Ct	6.36	7.1	6.23
**P value**	**0.1**		**0.001**
	**0.001**		

*Ct*, cycle threshold value in real-time PCR.

After the first step (overnight proteinase K digestion and lysis buffer), the semi-automated protocol took from 15 to 30 min without the intervention of an operator, compared to 3 hours for the manual technique, depending on the instruction of an operator. The automated protocol is performed into two steps, proteinase K digestion at 70°C for 10 min and the automated step for 15 min.

These data indicated that combining mechanical agitation in the presence of glass beads with enzymatic and chemical lysis significantly increased the yield of PCR-amplifiable archaeal DNA. The exact mechanism of these procedures was not tested here, but our previous experience suggests that these procedures not only efficiently break the cell walls, thus liberating the archaeal DNA, but also decrease the effects of PCR inhibitors [15]. We found that it was possible to further combine this manual part of the procedure with automated DNA extraction, thus significantly decreasing the protocol turn-around time and rendering archaeal DNA extraction and detection amenable to a routine procedure.

This study revealed that the DNA extraction method used strongly affects the apparent gut diversity and microbial community structure, as observed by real-time PCR tests. Each DNA extraction method revealed a different prevalence of *M. smithii*. Currently, no available stool DNA extraction method [16,17] is optimized to effectively extract archaeal DNA, contrary to that reported for plants [18,19]. Before the publication of the protocol described by Dridi et al in 2011 [15], the prevalence of *M. smithii,* the dominant archaea in the human digestive tract, was reported to be 30%, but it was detected in 91%-95.7% of stool samples using this protocol. This protocol has thus significantly increased the ability to detect archaea in the human gut. It has also allowed the PCR-based detection of a fourth archaeal species in the human gut, *M. luminyensis,* and led to its isolation and description [3]. However, the diversity of archaea in the human gut remains poorly studied. The DNA extraction protocol provided here can improve the exploration of the intestinal microflora, specifically the archaeal community; the identification of new species will increase knowledge in this area and promote the investigation of the potential roles of archaeal species in human diseases [11,12,20] and their effects on the bacterial microflora that colonize the human gastrointestinal tract.

In conclusion, proteinase K digestion and glass powder crushing dramatically increase the yield of archaea DNA from human stool samples. A semi-automated protocol could be used for extracting total stool DNA for further PCR amplification.

## Competing interest

The two co-authors (SK and MD) are co-inventors of a patent N/Réf: H52 888 cas 13 FR (MD/SB 12.05.00329) for the DNA extraction protocol reported here.

## Authors’ contributions

SK, PR, and MBB designed and performed the analyses; SK and MD interpreted the data and wrote the manuscript. All authors read and approved the final manuscript.
